# Image-Based Experimental Measurement Techniques to Characterize Velocity Fields in Blood Microflows

**DOI:** 10.3389/fphys.2022.886675

**Published:** 2022-04-29

**Authors:** Andy Vinh Le, Marianne Fenech

**Affiliations:** ^1^ Department of Mechanical Engineering, University of Ottawa, Ottawa, ON, Canada; ^2^ Centre de Biochimie Structurale, CNRS UMR 5048—INSERM UMR 1054, University of Montpellier, Montpellier, France

**Keywords:** microcirculation, kymographs, micro-particle velocimetry, dual-slit photometry, cell tracking

## Abstract

Predicting blood microflow in both simple and complex geometries is challenging because of the composition and behavior of the blood at microscale. However, characterization of the velocity in microchannels is the key for gaining insights into cellular interactions at the microscale, mechanisms of diseases, and efficacy of therapeutic solutions. Image-based measurement techniques are a subset of methods for measuring the local flow velocity that typically utilize tracer particles for flow visualization. In the most basic form, a high-speed camera and microscope setup are the only requirements for data acquisition; however, the development of image processing algorithms and equipment has made current image-based techniques more sophisticated. This mini review aims to provide a succinct and accessible overview of image-based experimental measurement techniques to characterize the velocity field of blood microflow. The following techniques are introduced: cell tracking velocimetry, kymographs, micro-particle velocimetry, and dual-slit photometry as entry techniques for measuring various velocity fields either *in vivo* or *in vitro*.

## Introduction

Microcirculation, which is composed of numerous vessels with diameters typically less than 50 μm, is the main exchange site between blood and tissues. Because blood behavior in the microcirculation cannot be considered as a liquid flow, and is rather regarded as a collection of interacting particles in suspension (Secomb, 2017), the study of blood flow in the microcirculation represents a considerable challenge. Red blood cells (RBCs), which are the most abundant cells found in the blood (40% of the blood volume), may adversely affect blood viscosity (Coull et al., 1991; Baskurt and Meiselman, 2007; Silva-Herdade et al., 2016; Sloop et al., 2020). For instance, blood viscosity increases when blood cells are too abundant (as shown in Erythrocytosis (Pearson and Path, 2001) and hyperleukocytic leukemia (Sharma et al., 1992), less deformable than healthy cells (sickle cell disorders (Connes et al., 2016), hemolytic anemia (Johnson and Ravindranath, 1996), and falciparum anemia (Parrow et al., 2018)), or if they tend to aggregate abnormally (observed in a variety of clinical states such as burns (Levin and Egorihina, 2011), infections and sepsis (Baskurt and Meiselman, 2013), complicated diabetes mellitus (Le Devehat et al., 1990), and malignant and rheumatic diseases (Luquita et al., 2009)). Velocity characterization is a key measurement in numerous studies, such as those analyzing the impact on microcirculation on cellular interactions due to aggregation, blood cell congestion, or platelet migration, to provide useful insights into the mechanisms of disease or to study therapeutic solutions (Bishop et al., 2001a; Long et al., 2004; Ishikawa et al., 2011; Kaliviotis et al., 2011; Sherwood et al., 2012; Pitts and Fenech, 2013b; Kaliviotis et al., 2018; Passos et al., 2019). The advent of microfluidics technology has propelled *in vitro* studies pertaining to the flow behavior of blood or blood cells in microchannels and the development of blood lab-on-a-chip platforms (Khalid et al., 2017; Maria et al., 2017; Sebastian and Dittrich, 2018; Passos et al., 2019; Abay et al., 2020; Mantegazza et al., 2020; Kihm et al., 2021).

Blood microcirculation studies are performed either *in vivo*, using intra-vital microscopy, or in a more controlled *in vitro* environment, taking advantage of the recent development and accessibility of microfluidic chips. The methods described here are image-based measurement techniques that are used in either *in vivo* or *in vitro* context, or are sometimes used in both contexts. Over the years, with the increase in the camera sampling frequency, advances in image processing techniques, and increase in computer calculation capacity, the original image-based method (cell tracking) has evolved into more complex methods that coexist and evolve in parallel. Although the choice of the method seems to be linked to the research community (*in vivo* vs *in vitro*) or to the experience of the research group and available equipment, this mini review aims to better guide the choice of a suitable method for a desired application by highlighting the capabilities and limitations of different methods. Table 1 summarizes key information as a quick guide.

**TABLE 1:** Comparison of image-based experimental measurement techniques to characterize velocity fields in blood microflows.

	Cell tracking	Kymograph	Micro-PIV	Dual slit
Tracers	Cells	Speckles from RBC shadows	RBCs or fluorescent microparticles	Speckles from RBC shadows
Image processing algorithms	See note	Kymograph	Cross-correlation	Cross-correlation
Critical parameters	*dt*	Image stability, focus, requires a flat (2-dimensional) microcirculation bed	DOC, *dt*, size and shape of CCW	DOC, relative position of the slits, slits shape
Hematocrit	<1%	Not reported	<20%	Not reported
Provide: characteristic velocity	yes	yes	yes	yes
Provide: velocity profile	yes	no	yes	yes
Provide: 2D velocity field in the vessel	yes	no	yes	yes
Provide: 3D velocity field in the vessel	no	no	yes, e.g., using confocal microscopy	no
Associated blood structural characteristics commonly explored in conjunction with velocity field	Aggregate size	Cell depleted layer	Aggregate size	
	Spatial distribution of red blood cells	Microcirculation velocity distribution	Hematocrit Profile	
	Cell depleted layer		Cell depleted layer	
	Viscosity Distribution		Viscosity Distribution	
Possibility to investigate unsteady flow	yes	Limited	limited	no
Typical Experimental equipment	a high-speed camera, a microscope	Kymograph only: a high-speed camera, a microscope	a light sensitive and short interframe times camera (double frame or high speed), a double pulsed laser with appropriate beam coupling and guiding, an epifluorescence microscope (single channel or stereo)	Photosensor (photodiodes, phototransistors, optical fibers) or a high-speed camera, a microscope
		*In vivo*: a portable high-speed camera with darkfield capacity coupled with an appropriate lens and a sterile disposable cap		

Note: Examples of cell tracking algorithms (not necessary RBC): multiple-hypothesis tracking of extracted cell barycenters, local optimization using a cost function within spatially limited search regions, state-space diagram optimization in a greedy fashion, nearest-neighbor tracking of extracted centers of mass, and iterative spatial-temporal association based on three-dimensional connectivity for 2D data (Maška et al., 2014).

## *In vivo* vs *in vitro* Studies

Numerous methods, such as Doppler, thermodilution, and dye injection, have been used to investigate microvascular flows (Gutterman et al., 2016; Secomb, 2017). However, here, we will focus only on image-based methods.

Intravital microscopy involves the observation of phenomena occurring in living organisms through a microscope. The simplest method is to operate on a small animal and exteriorize an organ, usually in the peritoneum, to be able to image it, which offers visualization of a flat microcirculatory network. The organ is immersed in a saline solution and immobilized with stitches (Bishop et al., 2001b; Long et al., 2004). Other research groups have opted for the implementation of a viewing window, which makes it possible to keep the animal alive for several days and to observe the spatial and temporal evolution of the microcirculation (Patumraj et al., 2005; Cabrales and Carvalho, 2010; Kihm et al., 2021).

Intravital microscopy images contain several artifacts that can influence velocity calculations, such as physiological movements, dilation and contraction of vessels, superposition of vessels in the organ, and light scattering. Open-source image processing tools are available to stabilize the images (e.g., the image stabilizer plugin for ImageJ (Li and Kang, 2008)).

**FIGURE 1:**
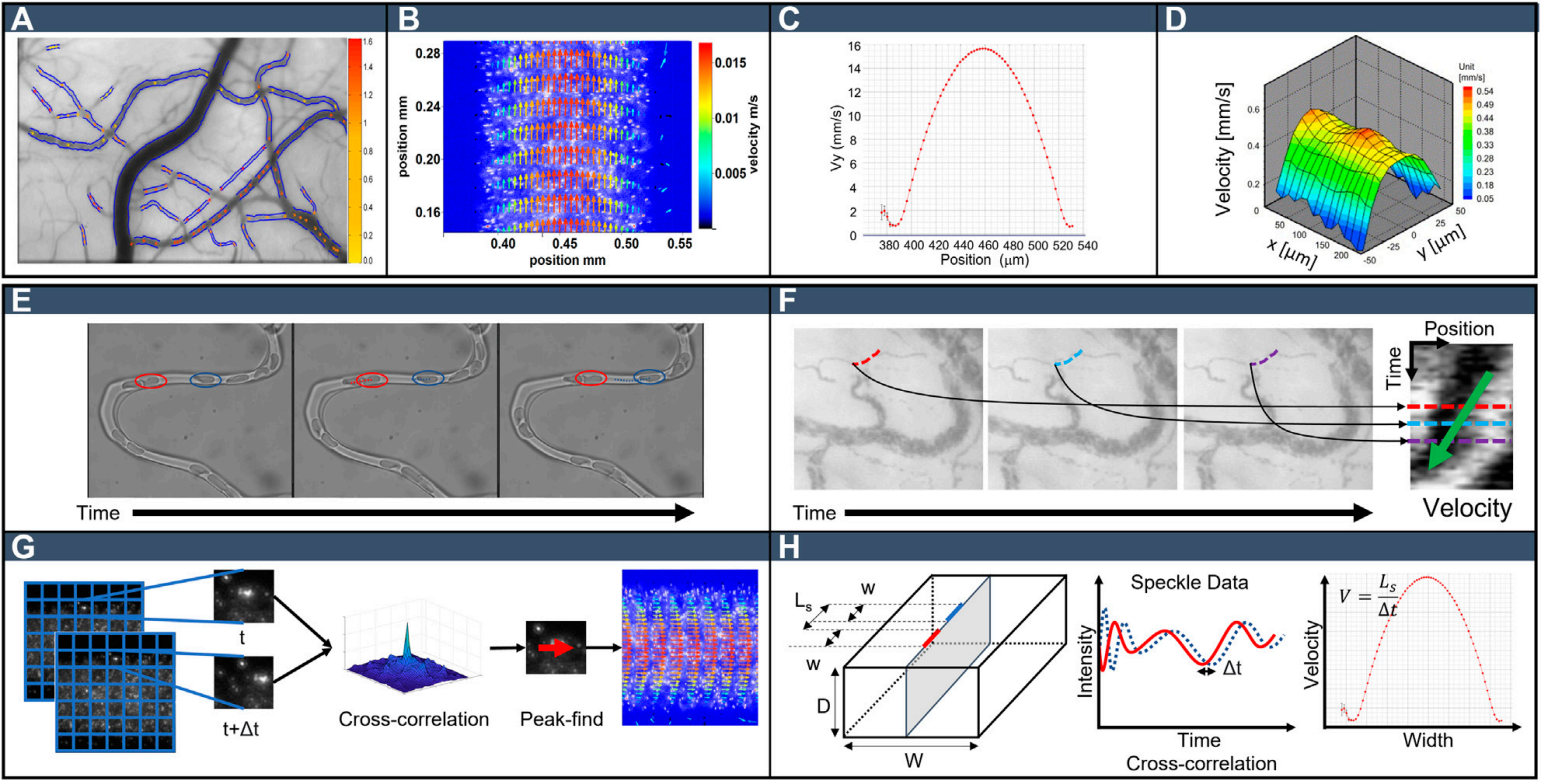
**(A-D)**: Visual comparison of typical velocity fields. **(A)** Color map of local velocity in a conjunctival microcirculation network. Color bar represents velocity in units of mm/s. The velocity vectors were obtained using kymographs image reused per United States government copyright (Khansari et al., 2015). **(B)** Field of 2D velocity vectors profile across a glass round 150 mm diameter capillary channel where blood, at 10% hematocrit, was flowing at a flow rate of 11.5 ml/min. The velocity vectors were obtained in our lab using micro-PIV. **(C)** Space-time averaged velocity corresponding to the field of 2D velocity vectors presenting in **(B)**. **(D)** Instantaneous 3D velocity profile of blood sample at 17% hematocrit in the central plane of a rectangular microchannel in in vitro analysis. Data were obtained using confocal micro-PIV, image reused with permission, copyright, 2022, Elsevier (Lima et al., 2007). **(E-H)**: Methods’ schematic of velocity determination **(E)** Cell tracking. The image presents *in vitro* RBCs in semi-circular curved channel **(F)** Kymograph. The image presents *in vivo* Sublingual microcirculation. **(G)** Micro-PIV. Images show fluorescent particles in the blood. **(H)** Dual slit photometry.

For *in vivo* studies, the velocity characterization methods aim for a characteristic velocity along each vessel (average velocity or maximum velocity), and the velocity field is then the map of velocities in the microcirculatory network, as shown in Figure 1A. In contrast, in *in vitro* studies on a single vessel, the methods seek to characterize the velocity profile along a line (Figure 1B) or velocity field in a cross-section (Figure 1C), and sometimes even the 3D velocity field (Figure 1D). *In vitro* studies, although less physiologically representative, offer the possibility of controlling parameters, such as geometry, flow rate, cell concentration, and pressure drop, while allowing better quantitative and qualitative velocity measurements.

All the methods presented here have a potential to be applied to *in vivo* and *in vitro* studies; however, typically, when red blood cell velocity is investigated in a single vessel, cell tracking velocimetry is used, while when a full network is to be analyzed, automatic vessel detection using the kymograph method is more popularly employed. For more detailed velocity field characterization, micro-particle image velocimetry (micro-PIV) and dual-slit methods are the reference methodologies; however, more advanced expertise is required.

## Cell Tracking Velocimetry

This technique involves identifying matching particles between the frames to link the positions. The velocities of the particles are then evaluated by dividing the time taken between each consecutive frame (Figure 1E). The first studies using cell tracking velocimetry were published in the 1960s (Bugliarello and Hayden, 1962). This method is simple to implement and captures temporal and spatial speed variations. It is mainly used *in vivo* and *ex-vivo*, but is also used *in vitro* (Omori et al., 2015). The first analysis was performed manually, but with the advent of computer automation, simple free access algorithms are available for quick analysis (Gonciarz, 2021). However, the simplicity of this method leads to several limitations. For instance, to be able to identify individual blood cells, the hematocrit, i.e., the RBC volume fraction, must be very low, or a small fraction of the cells must be fluorescently stained for tracking. RBC staining is a simple procedure that is easy to implement *in vitro*, and it has also been used *in vivo* in small animals (Horan et al., 1990; Asai et al., 2007; Agrawal et al., 2017). Only particles in the plane of interest (or focus) should be tracked; this can be done by visual inspection, however, automatic processing considers out-of-focus particles. In addition, RBCs can group due to aggregation, resulting in the tracked objects changing in size, disappearing due to overlapping, or changing in shape due to their rotation. These occurrences affect the accuracy of the automatic image processing (Niazi et al., 2019). Despite these limitations, cell tracking is still a very efficient and accurate method that is widely used to track the passage of single blood cells in microfluidics. A review regarding the comparison of different tracking methods was performed by Chenouard et al. (2014). Open source codes are available for simple tracking (e.g., (Tinevez et al., 2017; Gonciarz, 2021)). Some such codes include a position predictor (e.g., one based on a c) to account for cells that temporarily disappear, while more advanced tools integrate several popular machine learning algorithms to efficiently track objects from brightfield and fluorescence microscopy images (e.g., (39)).

## Kymographs

In addition to studying the velocity in a specific vessel, intravital microscopy is also used to investigate tissue perfusion under changing conditions. Typically, studies aim to capture the spatial and temporal variability of microcirculation affecting perfusion. In animal studies, all organs can be investigated, while in human studies, nail, coronary (Figure 1A), and sublingual microcirculation (Figure 1F) can be accessed non-invasively, while microcirculation at other sites, such as the brain, can also be investigated using endoscopies or during surgery.

Common imaging approaches utilize sublingual orthogonal polarization spectral (OPS) imaging, side-stream dark field (SDF) imaging, and incident dark-field (IDF) imaging (Dobbe et al., 2008; Massey and Shapiro, 2016) to record videos of microcirculation activity with enhanced contrast. In either case, high-speed image collection, typically >60 frames per second (fps), is required. For intravital microscopy, image stabilization is the first step in image analysis because of physiologically induced motions such as respiration and cardiac activity. Thus, vessel segmentation is necessary to identify the vasculature as shown in Figure 1A. Finally, the RBC velocities are determined using space–time diagrams, called kymographs, for every time interval and for every vascular segment. An example of a kymograph is shown in Figure 1F. A kymograph is obtained by plotting the intensity in the grayscale level along the vessel segment over time. On this new “image,” where space is encoded along the *x*-axis while time is plotted along the *y*-axis, velocity is then deduced from red blood cell shadow (speckles) angles (Jähne, 2005).

Typically, flow characterizations are presented as a color-coded velocity map, as illustrated in Figure 1A. Few commercial software packages are available to complete the full procedure (image acquisition, stabilization, segmentation, and velocity characterization) such as AVA (Microvision Medical B.V., Amsterdam, Netherlands) and CapiScope (KK Research Technology Ltd., Devon, England). The procedure was also implemented in the open source image software ImageJ under the name STAFF (Clendenon et al., 2019a; Clendenon et al., 2019b).

The method is highly dependent on the image quality, particularly image contrast and image sharpness. While illumination techniques have improved, the sharpness of the image is still an important limiting factor because the focus may be difficult to obtain when adjusting the lens on soft tissues and on living subjects, especially during bedside techniques, such as sublingual microcirculation, when the patient is awake. Companies manufacturing commercial acquisition devices, such as USB3 MicroScan (Microvision Medical B.V., Amsterdam, Netherlands), are still improving their focus-monitoring options.

## Micro Particle Image Velocimetry

Micro-PIV has been developed to determine the velocity field in microfluidics (Santiago et al., 1998), and since 2000, it has been used to characterize the velocity profiles of blood flowing through micro-channels (Okuda et al., 2003; Koutsiaris and Pogiatzi, 2004; Bitsch et al., 2005; Lima et al., 2006; Pasias et al., 2020). This technique is a particle-based flow visualization method. Figure 1G presents the schematic of the method. Under a microscope, two sequential images of the microflow are recorded at a known time interval (*dt*) at a fixed position. The pairs of images are used to determine the movement of a group of particles from the first image in the pair to the second image using cross-correlation within sub-regions known as cross-correlation windows (CCW). An interrogation window in the first image is compared within an interrogation window grid in the second image to find the most look-alike windows. The distance between the centers of both windows can be divided by the time elapsed between each frame to calculate the mean velocity of the particles for this position. To capture the displacement of the particles in a micrometric field of view, *dt* is set in conjunction with the size of the CCW. The average particle displacement between consecutive frames should be approximately a quarter of the size of the CCW (Scharnowski and Kähler, 2020) (from ms to μs depending on the lens magnification and flow velocity). This is a critical parameter because if *dt* is too large, the particles of the first image could pass beyond the second CCW, resulting in a loss of correlation.

Because this method is a particle-based technique, tracer particles are required. *In vivo* vs *in vitro* applications of micro-PIV require different equipment and execution choices. Traditionally, *in vivo* applications require high-speed photography using the RBCs themselves as tracers (Sugii et al., 2002; Koutsiaris and Pogiatzi, 2004; Bitsch et al., 2005; Lee et al., 2007; Kikuchi and Mochizuki, 2011), while in *in vitro* studies, artificial micro-fluorescent particles are added to the blood (Okuda et al., 2003; Lima et al., 2006; Lima et al., 2007; Lima et al., 2008; Pitts et al., 2012; Pitts and Fenech, 2013a; Mehri et al., 2015; Mehri et al., 2018; Pasias et al., 2020). Artificial tracer particles have also been used in living subjects in the chicken vitelline network, and embryonic heart (Poelma et al., 2008). Obtaining three-dimensional profiles of cell suspension flow is also possible by coupling micro-PIV with confocal microscopy (Lima et al., 2006). Micro-PIV offers a robust and accurate tool to characterize micro blood flows in 2D or 3D; however, the main disadvantage of this technique is the limitation in cell concentration, as the accuracy drops for RBC concentrations above 20% as the ability to resolve the particles decreases.

## Dual Slit Photometry

Dual-slit photometry is a technique developed by Wayland and Johnson (Wayland and Johnson, 1967) and is used to estimate the flow velocity and volumetric flow rates in microchannels (Lee et al., 1983). With optimized settings, it can be used to measure maximal velocity in microchannels both *in vivo* and *in vitro* (Roman et al., 2012b). The dual slit is a photometric technique that utilizes two photo sensors (slits) positioned along the vessel. The photosensors can be either photodiode sensors or are taken as two regions of interest in a digitized image. In order to obtain a full velocity profile, the slits’ width could be selected to be as small as 1 to 3 pixels, allowing for multiple velocity measurements along the cross-section of the channel (Roman et al., 2012a). Figure 1H presents the schematic of the method. In each slit, light fluctuations (speckles) are produced by the passage of the RBC flowing through the vessel. A time-dependent signal is captured for each slit in which the time delay between the two signals is obtained by temporal cross-correlation. The RBC velocities are then estimated by assessing the distance between the two slits.

Owing to the large number of RBCs in the channel, the difference in contrast between the cells and suspension media must be sharp to provide a good signal. Furthermore, the RBC rotation, shape, and migration in channels with larger diameters can result in two different signal outputs, preventing an accurate estimation of the velocity profile. For optimized measurement, the technique should be applied to vessels or microchannels that are linear where the slits can be placed at 5 to 10 particle size apart. Finally, the flow through the vessel must be steady, as the technique requires time averaging to ensure statistical convergence of the temporal cross-correlation (Roman et al., 2012b).

## Emergent Methods

Conventional methods to infer fluid velocity and pressure measurements or stress fields rely on either analyzing visual images as seen in the optical velocimetry techniques or by enforcing underlying physics without considering the visual data as completed in computational fluid dynamics (CFD). However, with the introduction of a physics-informed neural network, flow visualization image data, such as particles or injected-dye, can be used to derive quantitative information of the flow field while respecting any laws of physics described by a general partial differential equations (PDE) (Raissi et al., 2019). By encoding the Navier-Stokes equations into a neural network, the technique has been applied to flow visualization of both classical fluid problems and more complex flows such as the flow through a microaneurysm-on-a-chip (Raissi et al., 2019; Raissi et al., 2020; Cai et al., 2021). The steps for developing a physics-informed neural network include defining the underlying physical laws, configuring image data to be fed into the neural network, constructing the neural network architecture, training the neural network, verifying the robustness of the model, and finally deploying the model (Raissi et al., 2020; Cai et al., 2021).

The advantage of using a physics-informed neural network is its key property that it can be effectively trained using small datasets (Raissi et al., 2019). Furthermore, with 2D images from microfluidic experiments or from *in vivo* observations, a full estimate of the 3D velocity, pressure, and stress field of the system can be deduced, which may be challenging to measure experimentally or derive from the images alone (Raissi et al., 2019; Raissi et al., 2020; Cai et al., 2021). In contrast to traditional CFD methods, which are sensitive to boundary conditions, a physics-informed neural network can infer the flow field with the sequence of recorded data without any *a priori* knowledge of the inlet and outlet conditions. The implementation of a physics-informed neural network may also be considered more accessible as it can be implemented with open-source software such as TensorFlow (Abadi et al., 2016) on a powerful workstation or a local cluster (Raissi et al., 2020; Cai et al., 2021). However, the cost of utilizing a neural network for analysis is the computational cost of the development and training of the model itself, which is more computationally costly compared to simpler conventional optical methodologies relying only on visual images.

## Discussion

Table 1 presents a comparison of different methods and important parameters to inform decisions related to the method to be chosen for a specific study.

### Tracer Particles

Image-based experimental measurement techniques to characterize the velocity field of blood microflow utilize some form of tracer particles to visualize the flow. *In vivo*, natural tracers from the cells themselves can be used, while *in vitro* there are more options available for tracer selection from introducing microbeads, fluorescent molecules, or even marking the cells in the fluid sample. The use of RBCs has the advantage of not requiring particle injection and, more importantly, it avoids confounding interpretation attributed to the modification of the system under study. The use of RBCs as imaging tracers has nevertheless some disadvantages because their diameter is approximately 7 μm, while the diameter of an artificial fluorescent particle is typically on the order of 1 µm. The higher RBC particle size and density are responsible for the decrease in accuracy of the measurement method, increasing the depth of correlation (DOC), i.e., the depth at which "the correlation signal of particles significantly contributes to the correlation function” (Kloostermann et al., 2009; Nguyen et al., 2010; Chayer et al., 2012; Pitts and Fenech, 2013a). To minimize the effects of out-of-focus particles, “image-overlapping post-processing” method has proven to be beneficial specifically for blood micro-PIV (Pitts et al., 2012). When it is not possible to add tracer particles, it was shown that using RBCs as tracer particles has acceptable accuracy when analyzing for the value of the flow rate, but not when assessing for the specific shape of the velocity profile (Chayer et al., 2012; Pitts and Fenech, 2013a).

### Volume Fraction and Cell Contrast

Image-based techniques are severely limited by the feeding hematocrit of the system; it is difficult to distinguish cells or tracer particles introduced into the fluid medium in high hematocrit studies. This effectively limits the study of the dynamics of blood microflows using imaging techniques, as the accuracy of measurements decreases as the hematocrit approaches values closer to the physiological values. The volume fraction is a significant factor to consider when selecting an optical measurement technique because if the cell concentration is too high, automatic cell tracking and PIV algorithms are less accurate, while the contrast of the cells can affect the accuracy of kymography and dual-slit photometry. Measuring flow fields in dense suspension of cells would require techniques that utilize sensors that can penetrate denser samples, such as ultrasound localization microscopy (Errico et al., 2015) or those with modifications to RBC opacity, such as converting RBCs into ghost cells (Jansen et al., 2016).

### Type of Velocity Field

To extract a characteristic velocity, such as the mean or max velocity, any technique presented here can be utilized. However, to expand into the 2D velocity field of the vessel, cell tracking, micro-PIV, and dual-slit photometry should be considered; in micro-PIV, it is also possible to obtain 3D profiles with additional equipment. With the most basic equipment, the techniques are not necessarily independent and differ by the image processing algorithms that are applied to the acquired images; however, the optimal setting of certain factors, such as the channel configuration, lighting, and lens type, can drastically change the accuracy of the algorithm applied. To obtain more detailed velocity profiles in 3D or under a broad range of flowrates would require more sophisticated equipment such as cameras with higher acquisition rates, pulsed lasers, or filters. This can make profiling of the velocity field in blood microflow an expensive endeavor because the upfront cost of the equipment can be high.
